# Ureteroinguinal Herniation with Consecutive Ureteral Stricture in a 2-Month-Old Infant: Case Report

**DOI:** 10.1055/s-0044-1779253

**Published:** 2024-01-22

**Authors:** Carlos Delgado-Miguel, Antonio Jesus Muñoz-Serrano, Pablo Aguado, Ennio Fuentes, Ricardo Díez

**Affiliations:** 1 Department of Pediatric Surgery, Hospital Universitario La Paz, Madrid, Spain; 2 Department of Pediatric Surgery, Hospital Universitario Fundacion Jimenez Diaz, Madrid, Spain

**Keywords:** ureteroinguinal hernia, herniation of the ureter, hydronephrosis, upper urinary tract obstruction

## Abstract

Inguinal herniation of ureter is an uncommon finding among children, with scarce reported cases in the literature to date, that can potentially lead to obstructive uropathy. We report a case of ureteroinguinal herniation discovered during an inguinal hernia repair in a patient with antenatally ultrasound finding of hydronephrosis. A 2-month-old infant with antenatal left hydronephrosis presented with left inguinal mass. Preoperative ultrasound showed an anechoic tubular image producing a mass effect on the left testicle, with suspected bladder herniation and/or dilated ureter toward the inguinal canal. An open surgical inguinal exploration was performed, where the left inguinal canal revealed a peritoneal sac and sliding of the dilated left ureter behind the sac, with a significant change in diameter, corresponding to the paraperitoneal variant of ureteroinguinal herniation. Ligation of the sac and replacement of the ureter into the retroperitoneum were performed, with improvement in the hydronephrosis observed on the ultrasound 1 month after the intervention. However, 6 months later, hydronephrosis worsening as well as the obstructive pattern observed in the diuretic renogram required removal of the stenotic ureteral segment and reimplantation of the healthy proximal segment in the bladder by open approach (Cohen's reimplantation). Follow-up ultrasound of the renal tract showed no dilatation of the upper renal tract and the renal function tests were normal. Currently, the patient is 2 years old and he remains asymptomatic. In conclusion, **s** igns of ureteral obstruction such as hydronephrosis in patients with inguinal herniation may suggest the possibility of an ureteroinguinal hernia. Preoperative diagnostic suspicion is essential.

## Introduction

Inguinal herniation of ureter is an uncommon finding among adults and even more so in children that can potentially lead to obstructive uropathy. 1 While inguinal hernias involving the bladder are well described, ureter involvement remains an uncommon phenomenon, as it is a retroperitoneal structure. 2 There have been over 140 reported cases in adults, but only a few concerning pediatric patients. 3 We report a case of inguinal herniation of ureter discovered during an inguinal hernia repair in a patient with antenatally hydronephrosis ultrasound finding.

## Case Presentation

A male patient with antenatal left hydronephrosis, in whom an inguinal mass was discovered by the parents incidentally during bathing at 2 months of age. Examination revealed a reducible mass in the left inguinal region. Remaining physical examination was unremarkable, with both testes found in the scrotum. Preoperative ultrasound showed an anechoic tubular image producing a mass effect on the left testicle, with suspected bladder herniation and/or dilated ureter toward the inguinal canal.

**Fig. 1:**
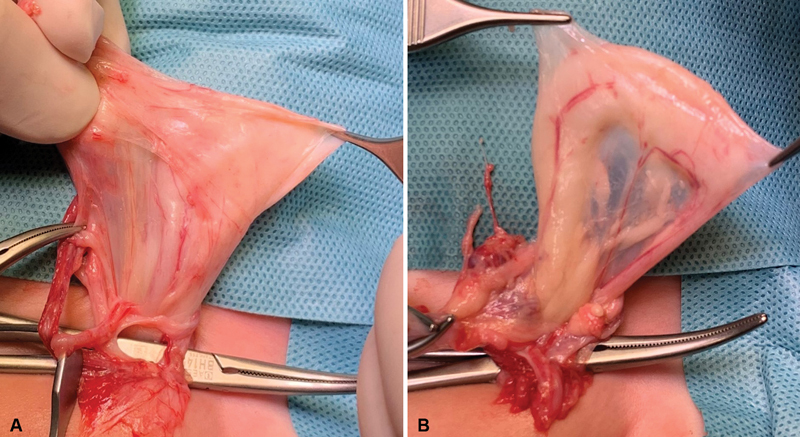
Intraoperative images. ( **A** ) Hernia sac with sliding of the dilated left ureter behind the sac. ( **B** ) Significant change in ureteral diameter after hernia sac ligation.

An open surgical inguinal exploration was performed, where the left inguinal canal revealed a peritoneal sac and sliding of the dilated left ureter behind the sac, with a significant change in diameter, corresponding to the paraperitoneal variant of ureteroinguinal herniation ( Fig. 1A ). The ureter emanated from the retroperitoneum and a loop lay inside the inguinal canal. Kinking and partial obstruction occurred at the level of the structure, leading to proximal dilation ( Fig. 1B ). Ligation of the sac and replacement of the ureter into the retroperitoneum was performed. The patient was discharged the same day, few hours after surgery, without postoperative complications. One month after the intervention, an improvement in the hydronephrosis was observed on the ultrasound, so we decided to manage it conservatively, maintaining the antibiotic prophylaxis (amoxicillin), which had been started after birth. Renal scintigraphy showed no differences in size or renal function between the two kidneys (right kidney: 49.8%, 6.1 × 2.8 cm; and left kidney: 50.2%, 6.3 × 4 cm).

However, at 6 months postoperatively, a worsening of hydronephrosis was observed on ultrasound examination. Diuretic renogram showed a delayed arrival of the radiotracer on the left side compared with the right side, with a dilated middle and proximal third of the left ureter compatible with ureteral obstruction. Due to these findings, an elective resection of the stenotic left ureter (5 cm in length) with remodeling and ureteral reimplantation according to Cohen's technique was performed using the Pfannenstiel approach. A double J stent was placed. The patient was discharged the following day. Six months later, after verifying complete correction of the hydronephrosis by ultrasound, double J stent was removed without incident. Follow-up ultrasound of the renal tract after double J stent removal showed no dilatation of the upper renal tract and the renal function tests were normal. Currently, the patient is 2 years old and he remains asymptomatic. We obtained the approval of the institutional ethics committee and the hospital review board (PI-6410). Written informed consent was obtain from parents of the patient to publish this study.

## Discussion

This case and literature review provide valuable insight into preoperative diagnostic difficulties, as well as the intra- and postoperative management of ureteroinguinal hernia in children, highlighting the importance of accurate diagnosis, close follow-up, and appropriate surgical intervention associated with this disease. While most cases of inguinal ureteral herniation have been reported in adults, only a few cases have been reported in children, 4 5 6 7 8 9 10 11 12 13 interestingly all of them in boys, with no description of this entity in girls to date. The median age reported at diagnosis was 4 months of age (range: 1 month–14 years), which is uncommon in children older than 18 months. Only two cases have been reported in adolescents, one with a past medical history of left inguinal hernia surgery when he was 1 year old, and the other case with a complex malformation (cloacal exstrophy). 10 11

There are two variants of ureteroinguinal hernias based on their anatomical structure: the paraperitoneal type (80%), with a true hernia sac that drags the ureter into the inguinal canal via traction, and the extraperitoneal type (20%), which does not have an associated hernia sac and is believed to be related to abnormal development of the urinary system, such as abnormal development of the Wolffian duct and adhesion between the primitive ureter and the genitofemoral ligaments. 14 Indeed, many cases of extraperitoneal hernias are associated with malformations of the kidney and urinary tract, such as wandering kidney and crossed renal ectopia, while paraperitoneal types are more commonly associated with vesicoureteral reflux, posterior urethral valve, giant ureter, and polycystic dysplastic kidney. 15 In this aspect, our case is atypical as it presents a ureteroinguinal herniation with no other associated renal anomalies.

In both types, there are often no specific symptoms other than distention of the inguinal region due to slipping of the ureter. As in our case, children with ureteral hernias rarely have any urinary symptoms and usually present with a mass in the inguinal o scrotal area. Symptoms owing to obstructive uropathy are not frequently seen, with hydronephrosis being described in only two of the other nine case reports. Most patients are asymptomatic, and routine urographic studies are usually not obtained preoperatively in these patients. For this reason, the diagnosis of ureteroinguinal hernia is often missed until surgical exploration. 4 However, if signs of ureteral obstruction, such as hydronephrosis or hydroureter, are observed in cases of inguinal herniation, the possibility of an inguinal ureteral hernia should be considered. Hydronephrosis may be secondary to angulation of the ureter or due to direct ureteral compression because of inflammation of other herniated contents. 10 In our case, the prenatal diagnosis of left hydronephrosis and the presence of the inguinoscrotal bulge on the same side put us on alert and we requested a scrotal ultrasound prior to surgery, which is not routinely performed in most children with suspected inguinal hernia. Other authors have reported preoperative intravenous urography or micturating cystourethrogram, 8 although these imaging tests involve ionizing radiation.

After diagnosis confirmation during surgical exploration, there are different treatment options depending on the individual appearance of the herniated loop of ureter. If there is no ureteral injury, most authors have repositioned the herniated ureters back into the retroperitoneum along with hernial repair. 3 6 9 11 However, in case of questionable viability, injury or severe dilated ureters, partial resection and anastomosis should be performed. 10 Temporary ureterocutaneostomy has been reported for extremely dilated ureters, with the intention of later reimplantation. 7 8 In cases with widespread ureteral involvement, ureteric repair can also be performed using transureteroureterostomy and appendiceal interposition. 4 In our case, although there was a significant change in ureteral caliber, we assumed that the ureteral kinking within the inguinal hernia was responsible for the dilatation of the proximal ureter and hydronephrosis due to the extrinsic compression, so the ureter was reintroduced into the abdominal cavity without performing any other associated surgical procedure. The presence of the ureter inside the hernia may had probably occurred during pregnancy, which could explain the diameter change observed. Due to the good appearance of the ureter during surgery, well perfused, and not ischemic, we decided to perform conservative management and give an option for resolution of the hydronephrosis after correction of the hernia and repositioning of the ureter in the retroperitoneum. Postoperative monitoring for urinary tract obstruction is important in these patients, due to the risk of pyelonephritis or worsening of hydronephrosis. 12 Regular ultrasound scans are essential during follow-up, as they provide information on the dilatation of the urinary tract. Preventing pyelonephritis and subsequent renal scarring is essential in patients with ureterohydronephrosis, in whom antibiotic prophylaxis should be started soon after diagnosis. 13 16 In the case of breakthrough infections, early surgical intervention is required to prevent renal damage in the long term. 1 In our patient, the transient improvement in hydronephrosis after surgery allowed us to continue conservative management. However, hydronephrosis worsening 6 months after surgery and the obstructive pattern observed in the diuretic renogram required removal of the stenotic ureteral segment and subsequently healthy proximal segment reimplantation. Although ureteral release from the inguinal canal allowed an initial urinary tract dilatation improvement, the fibrosis of the stenotic ureteral segment required resection and reimplantation for definitive resolution of the hydronephrosis.

Laparoscopic approach in these patients is controversial. Although it may provide better visualization of the ureter entering the inguinal canal, hernia repair may require an open approach, as reported by Cianci et al. 11 In our case, laparoscopic approach would have determined the ureter entering the inguinal canal, next to the hernial sac, corresponding to the paraperitoneal variant of ureteroinguinal hernia, but ureteral dissection would have been more difficult and with less vascular control than with open surgery. Due to the preoperative suspicion of ureteroinguinal hernia, we performed a very careful inguinal dissection to avoid ureteral injury.

## Conclusion

Even though ureteral herniation in infants occurs sporadically, it is important to recognize to prevent ureteral damage during inguinal hernia repair or, as was described here, as it may lead to obstructive nephropathy. If there is a recognized urinary tract anomaly associated ipsilaterally as the hernia such as hydronephrosis, then ureteroinguinal hernia should be sought for preoperatively. Conversely, if one encounters ureteroinguinal hernia during routine herniotomy, an associated urinary tract anomaly should also be ruled out.
